# Sleep characteristics before assisted reproductive technology treatment predict reproductive outcomes: a prospective cohort study of Chinese infertile women

**DOI:** 10.3389/fendo.2023.1178396

**Published:** 2023-10-16

**Authors:** Qian-Ling Li, Chao Wang, Ke-Xin Cao, Lin Zhang, Yun-Shuai Xu, Liang Chang, Zhen-Hui Liu, Ai-Jun Yang, Yan-Xue Xue

**Affiliations:** ^1^ National Institute on Drug Dependence and Beijing Key Laboratory of Drug Dependence, Peking University, Beijing, China; ^2^ Center for Reproductive Medicine, Affiliated Hospital of Jining Medical University, Jining, China; ^3^ Reproductive Medical Center, Department of Obstetrics and Gynecology, Peking University International Hospital, Beijing, China; ^4^ Department of Pharmacology, School of Basic Medical Sciences, Peking University, Beijing, China; ^5^ Department of Psychiatry, Shandong Daizhuang Hospital, Jining, China; ^6^ Henan Key Laboratory of Medical Tissue Regeneration, School of Basic Medical Sciences, Xinxiang Medical University, Xinxiang, China; ^7^ Chinese Institute for Brain Research, Beijing, China; ^8^ Key Laboratory for Neuroscience, Ministry of Education/National Health Commission, Peking University, Beijing, China

**Keywords:** sleep quality, infertile women, anxiety, depression, stress, positive affect, assisted reproductive technology (ART), reproductive outcomes

## Abstract

Sleep disorders affect mental and physical health. Infertile women undergoing assisted reproductive technology (ART) treatment are prone to sleep disorders. Sleep condition, its influencing factors, and the association between sleep condition and ART treatment outcomes before treatment have not been explored within a population with a large sample size. Therefore, we investigated the sleep characteristics of 1002 Chinese infertile women before ovulation induction and investigated the influencing factors (negative and positive psychological factors, demographics, and fertility characteristics). We also examined whether sleep conditions before treatment predicted reproductive outcomes. We found that 24.1% of participants reported poor sleep quality. Women with primary infertility reported poorer sleep than women with secondary infertility. Negative psychological factors, including depression, anxiety, and perceived stress were associated with poor sleep, whereas positive affect was linked with good sleep. Adverse sleep characteristics, including poor subjective sleep quality, sleep disturbances, and poor sleep efficiency, decreased the quantity and quality of oocytes retrieved, fertilization rates, and clinical pregnancy rates. This study indicates that before ART treatment, a large number of females with infertility suffer from sleep problems, which are affected by psychological factors and infertility type, and unhealthy sleep characteristics may impair treatment outcomes. Our findings highlight the importance of screening and treatment for sleep disorders before the enrollment of ART treatment in infertile women.

## Introduction

Sleep quality is an important factor that affects human health. Sleep disorders are associated with depression and anxiety, as well as an increased risk of cardiovascular disease, hypertension, diabetes (1–5), and infertility (6–10). Infertility is a widespread health problem. Infertility is a widespread global health problem. Infertility is a disease of the male or female reproductive system defined by the failure to achieve a pregnancy after 12 months or more of regular unprotected sexual intercourse (11). Worldwide, 10-15% of couples of childbearing age suffer from infertility (12). Assisted reproductive technology (ART) eases the burden of infertility on individuals and families. Until 2020, more than 8 million babies worldwide have been successfully born using techniques such as IVF/ICS (13). However, the overall clinical pregnancy rate maintained at only approximately 35% (14, 15). The success of infertility treatment depends mainly on the quality of the oocytes (16). In addition to physiological and drug factors, sleep quality can also affect oocyte quality (16). Chronic sleep deprivation is a biological stress (17) that enhances the activity of the hypothalamic-pituitary axis (18) and sympathetic nervous system (19) and leads to excessive oxidative stress (20–22). The increase in reactive oxygen species (ROS) in patients with sleep disorders interferes with the follicular microenvironment to a great extent and further affects the quality of follicles as well as the development and differentiation of embryos (16, 22), which may have a detrimental effect on ART treatment outcomes (23–27). Therefore, it is necessary to pay attention to sleep quality in women who plan to undergo IVF/ICSI.

Sleep disorders are prevalent in patients undergoing ART treatment (16, 28, 29). Sleep disorders include too short or too long sleep duration, sleep fragmentation, circadian dysrhythmia, and hypoxia (9). Sleep quality assessment with PSQI suggested that the detection rates of poor sleep quality in females before and during IVF/ICSI were different (29–32). Although the sleep quality of females undergoing IVF/ICSI treatment is gaining more interest, sleep status prior to treatment is rarely assessed. The sample sizes of previous studies that investigated the incidence of sleep disorders before IVF/ICSI treatment were relatively small (29, 32). In a study involving 21 participants, 57% of the participants had sleep disturbances before treatment (29). Another study involving 163 participants found that most participants (92.2%) reported poor sleep quality before treatment (32). During treatment, patients were given ovulation induction drugs and underwent invasive procedures (33). A study with a large sample size to investigate the sleep characteristics of women before ovulation induction will avoid the bias caused by the small sample size and control the confounding effects of pharmacotherapy.

Sleep quality and mental health are closely related. Irritability and anxiety affect the quality of sleep (34), and negative emotions and sleep disorders may form a vicious circle. Women experience more psychological stress than men during ART treatment (35–37), resulting in anxiety, depression, and other psychological disturbances (38, 39) and sleep disorders (31, 40). In addition, at different stages of IVF/ICSI treatment, the prevalence of anxiety and depression differ (30, 41). Although many studies have explored the psychological condition of females undergoing IVF, only a few studies have explored the psychological factors that impact sleep quality (30, 32, 42), and they mainly focus on negative psychological factors. Little is known about the association between positive psychological factors and sleep.

In this study, we collected data on sleep characteristics in women who planned to undergo IVF/ICSI and analyzed factors (negative and positive psychological factors and fertility characteristics) that may affect sleep quality. We also assessed the relationship between sleep characteristics and reproductive outcomes (the number of oocytes retrieved, the oocyte retrieval rate, the number of mature oocytes, the number of good-quality embryos, fertilization rate, and clinical pregnancy). We hypothesized that both negative and positive psychological factors contribute to sleep quality. We also hypothesized that sleep quality before ART treatment is associated with both intermediate reproductive outcomes and clinical pregnancy rate.

## Materials and methods

### Participants and study design

This is a prospective cohort study. From April 2020 to October 2021, we recruited a convenience sample of 1142 females who planned to undergo ART treatment at the Reproductive Medicine Department, Affiliated Hospital of Jining Medical University, China. Inclusion criteria: a. Participants who had regular sexual intercourse without contraception for at least one year without pregnancy (43); b. Participants who planned to undergo IVF/ICSI; c. Age ≥20 years; d. Participants must be able to complete the questionnaire independently; e. No other serious physical illness. Exclusion criteria: a. History of major life events in the last 2 months; b. History of major mental illness. Each participant gave written informed consent, and all protocols were approved by the Medical Science Ethics Board of Affiliated Hospital of Jining Medical University (2020C081).

**Figure 1:**
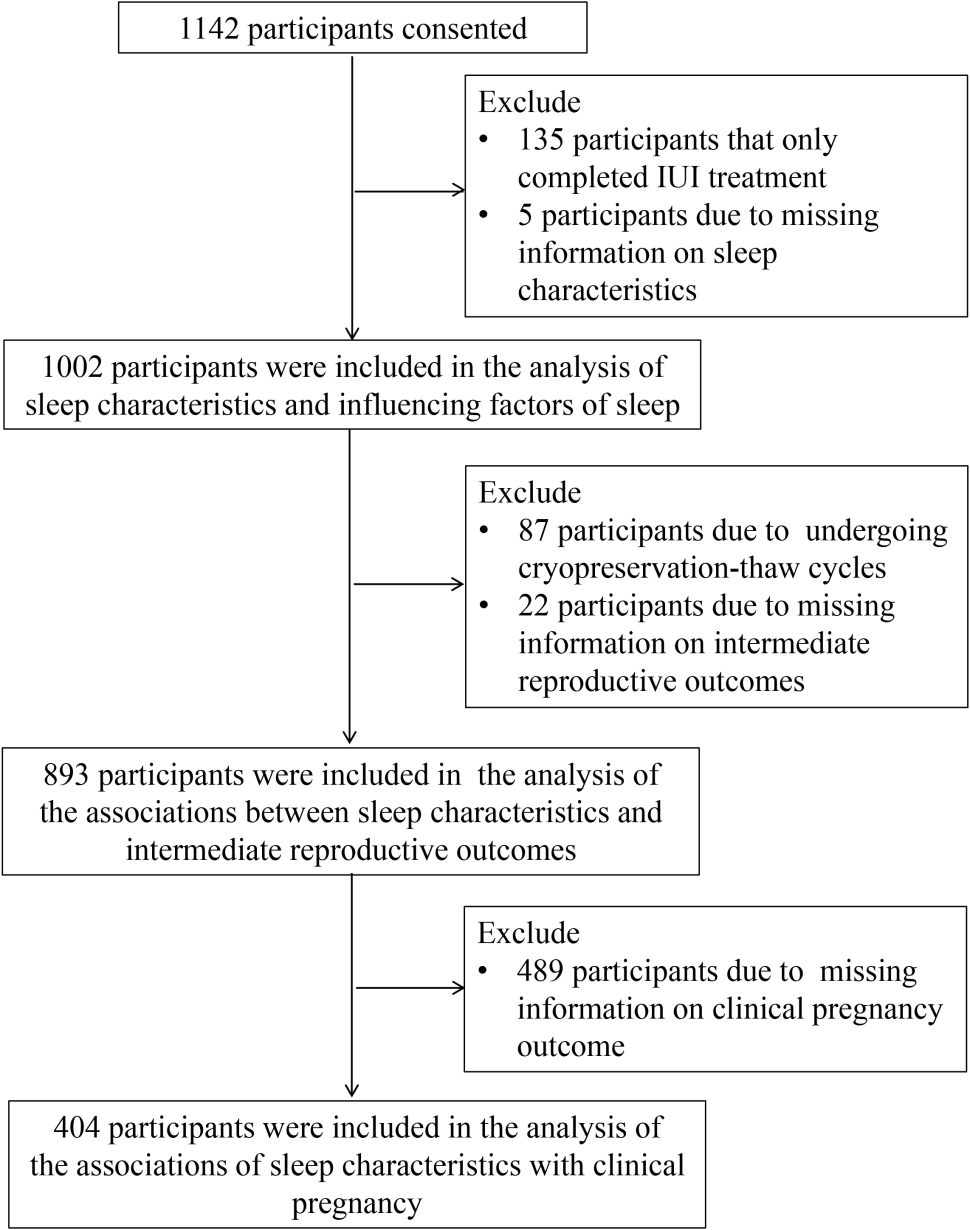
Analysis workflow, including the total number of participants for each analysis step.

At enrollment, each participant completed a questionnaire on demographic information and fertility. On the day of ovulation promotion of IVF/ICSI treatment, the participant received the study questionnaire. In the analysis of sleep characteristics and the influencing factors of sleep quality, 135 participants who completed IUI cycles and 5 participants who lacked information on sleep characteristics were excluded, and 1002 participants were included. In the analysis of the relationship between sleep characteristics and intermediate reproductive outcomes, women who experienced cryopreservation-thaw cycle (N = 87) and women who lacked intermediate clinical reproductive outcomes (N = 22) were further excluded, resulting in a total of 893 participants included. In analyzing the relationship between sleep characteristics and clinical pregnancy outcomes, women lacking information on clinical pregnancy outcomes of IVF/ICSI were further excluded (N = 489), and 404 participants were included **(Figure 1**).

### Data collection

The baseline questionnaire was used to collect data on sociodemographic characteristics and fertility status. Validated standardized scales were used to assess participants’ psychological states including depression, anxiety, perceived stress, and sleep quality prior to ART. IVF/ICSI treatment outcomes were extracted from the hospital information system, which included the number of oocytes retrieved, oocyte retrieval rate, number of mature oocytes, number of high-quality embryos, fertilization rate, and clinical pregnancy.

### Measures

#### Basic personal information questionnaire

The custom questionnaire collects demographic information such as age, body mass index, education level, economic status, and fertility information such as cycle type, duration of infertility, infertility type, cause of infertility, number of cycles, past pregnancy history, and previous treatment.

#### Pittsburgh Sleep Quality Index

Pittsburgh Sleep Quality Index (PSQI) was developed in 1989 by Buysse and colleagues and has been widely used to assess sleep quality (44, 45). PSQI consists of 19 items rated by self and 5 items rated by roommate or bedpartner. The 5 other-rated items were used for clinical information only and were not included in the scoring of PSQI. The 19 self-rated items are classified into seven components: subjective sleep quality, subjective sleep quality, sleep latency, sleep duration, habitual sleep efficiency, sleep disturbances, use of sleeping medication, and daytime dysfunction. Each component scores 0–3 and the scores of the seven components are added together to obtain a total PSQI score to measure global sleep quality. A cut-off score of > 5 was utilized, and the sensitivity and specificity were 89.6% and 86.5%, respectively (44).

#### Beck Depression Inventory-Short Form

Beck Depression Inventory-Short Form (BDI-13) is widely used to evaluate the severity of depression. BDI consists of 13 items, and each item scores 0–3. The scoring assessment is as follows: 0–4 no depression, 5–7 mild depression, 8–15 moderate depression, 16–39 severe depression (46).

#### Self-rating anxiety scale

Zung’s self-rating anxiety scale (SAS) assesses subjects’ subjective feelings of anxiety (47). The scale includes 20 items that cover 4 groups of manifestations: cognitive, autonomic, motor, and central nervous system symptoms. Each item is rated on a Likert-type scale of 1–4, and the final score was equal to the integer part of the total score of 20 items multiplied by 1.25. Anxiety standard scores ≥ 50 were considered to be at risk for clinical anxiety (48).

#### Perceived Stress Scale

Perceived Stress Scale was developed in 1983 by American psychologist Cohent and contains two dimensions: sense of loss of control and tension. Each item is scored on a 5-point scale and the total score ranges from 0 to 56, with higher scores indicating greater perceived stress. The Cronbach’s coefficient of the scale is 0.84–0.86, indicating good reliability (49, 50).

#### Connor-Davidson resilience scale

Connor-Davidson resilience scale (CD-RISC-25) was developed by American psychologists Connor and Davidson (51) and is one of the most widely used tools for measuring psychological resilience. The scale contains 5 factors: 1) notion of personal competence, high standards, and tenacity; 2) trust in one’s instincts, tolerance of negative affect, and strengthening effects of stress; 3) positive acceptance of change, and secure relationships; 4) control, and 5) spiritual influences. The scale is scored using a 5-point scale response. The total score ranges from 0 to 100, with higher scores reflecting better psychological resilience. The internal consistency reliability of the scale was 0.89 and the test-retest reliability was 0.87, and it had good convergent and discriminant validity (51).

### Statistical analysis

Data were organized using Excel 2016 and analyzed using SPSS 27.0. The presence or absence of sleep disturbances was used as the grouping variable. Normally distributed continuous variables were expressed as mean ± standard deviation and categorical data were expressed as frequency and proportion. Comparisons between good and poor sleepers were performed using independent samples t tests for continuous variables and χ2 or Wilcoxon rank sum test for categorical variables. Pearson correlation was used to measure the correlation between psychological factors and sleep characteristics. Multiple logistic regression analyses were used to assess the influencing factors of the sleep quality of participants who planned to undergo IVF/ICSI. We used a generalized linear model to assess the relationship between sleep characteristics and IVF/ICSI outcome. Sleep characteristics included subjective sleep quality, trouble falling asleep, nighttime sleep duration, sleep efficiency, sleep disturbance, and daytime dysfunction due to sleepiness, all of which are categorical variables. For the number of oocytes retrieved, the number of mature oocytes, and the number of high-quality embryos, a negative binomial distribution and log link function were specified. For oocyte retrieval rate and fertilization rate, a linear function was specified. For clinical pregnancy rate, a binary logistic regression was specified. To effectively control for confounders, the final model included age (continuous variables), BMI (continuous variables), duration of infertility (continuous variables), infertility type (primary *vs*. secondary), cause of infertility (female factors, male factors, mixed factors or unexplained), previous pregnancy (no *vs*. yes), and number of previous IVF/ICSI cycles (one *vs*. two or more). Our results reported incidence rate ratio (IRR)/odds ratios (OR) and 95% CI. IRR/OR < 1 indicates a reduced chance of the target event occurrence. The significance level was set at a two-tailed 5%. No mathematical correction was made for multiple comparisons.

## Results

### Demographics and fertility characteristics

We include 1002 participants. Their average age was 32.66 ± 5.06 years old. The average BMI was 23.50 ± 3.66 kg/m^2^. Six-hundred and thirty-three (62.9%) participants had a high school or higher diploma. The average infertility duration was 3.08 ± 2.34 years. Three-hundred and twenty-two (32.1%) participants had primary infertility. For details on demographic and fertility characteristics, see **Table 1**.

**Table 1:** Demographic and fertility characteristics (N = 1002).

Variables	Total, N = 1002	Good sleepers, N = 759	Poor sleepers, N = 243	p-value
Age (years)				
< 35	669 (66.8)	499 (65.8)	170 (70)	0.235
≥ 35	332 (33.2)	259 (34.2)	73 (30)	
BMI^a^ (kg/m^2^)	23.51 ± 3.66	23.36 ± 3.52	23.97 ± 4.06	0.036
Education level				
Junior high school or below	369 (36.9)	290 (38.3)	79 (32.5)	0.106
High school and above	632 (63.1)	468 (61.7)	164 (67.5)	
Income (yuan, per month)				
<1000	32 (3.2)	27 (3.6)	5 (2.1)	0.935
1000–2999	254 (25.4)	195 (25.7)	59 (24.3)	
3000–4999	420 (42)	309 (40.8)	111 (45.7)	
5000–9999	194 (19.4)	145 (19.1)	49 (20.2)	
> 10000	101 (10.1)	82 (10.8)	19 (7.8)	
Cycle type				
Freezing cycle	87 (8.9)	61 (8.2)	26 (10.9)	0.204
Fresh cycle	896 (91.1)	683 (91.8)	213 (89.1)	
Duration of infertility (years)				
< 5	790 (79.3)	595 (78.9)	195 (80.6)	0.578
≥ 5	206 (20.7)	159 (21.1)	47 (19.4)	
Infertility type				
Primary infertility	320 (32.6)	220 (29.6)	100 (41.8)	< 0.001
Secondary infertility	663 (67.4)	524 (70.4)	139 (58.2)	
Cause of infertility				
Male factor	190 (19)	140 (18.5)	50 (20.7)	0.648
Female factor	502 (50.2)	379 (50)	123 (50.8)	
Mixed factors	187 (18.7)	148 (19.5)	39 (16.1)	
Unexplained	121 (12.1)	91 (12)	30 (12.4)	
Previous pregnancy				
No	346 (34.5)	252 (33.2)	94 (38.7)	0.118
Yes	656 (65.5)	507 (66.8)	149 (61.3)	
Number of cycles				
One	881 (89.4)	664 (89)	217 (90.4)	0.539
Two or more	105 (10.6)	82 (11)	23 (9.6)	

a BMI: body mass index

### Global sleep quality and sleep characteristics before ovulation induction

Among the participants, 243 had poor sleep (PSQI > 5). BMI and infertility type of good and poor sleepers differed significantly. A higher BMI was associated with poorer sleep quality. The sleep quality of participants with primary infertility was worse than that of participants with secondary infertility. Age, household income, cycle type, duration of infertility, cause of infertility, previous pregnancy, and the number of previous cycles did not impact sleep quality (**Table 1**).

As for specific sleep characteristics, 6.2% of the participants reported poor or very bad subjective sleep quality, 9.5% reported a sleep latency of more than 30 minutes, 8.2% reported a sleep duration of fewer than 7 hours, and 23.9% reported a sleep efficiency lower than 85%. Most participants (99%) were not using sleeping medication. Approximately half (52.6%) of the participants reported daytime dysfunction (**Table 2**). Sixty-three women reported night shift experience.

**Table 2:** Sleep characteristics (N = 1002).

Characteristics	N	%
Subjective sleep quality		
Very good	348	34.7
Fairly good	592	59.1
Fairly bad	56	5.6
Very bad	6	0.6
Sleep latency		
(1) Time to fall asleep		
15 min	397	39.7
16–30 min	510	50.9
31–60 min	81	8.1
≥ 61 min	14	1.4
(2) Cannot fall asleep within 30 min		
Never	676	67.5
< 1/week	201	20.1
1–2/week	82	8.2
≥ 3/week	42	4.2
Sleep duration		
< 7 h	82	8.2
7 to < 8 h	282	28.2
8 to < 9 h	454	45.3
9 to < 10 h	124	12.4
≥ 10 h	60	6
Habitual sleep efficiency		
85%	763	76.1
75–84.9%	180	18
65–74.9%	47	4.7
≤ 64.9%	12	1.2
Sleep disturbances		
No	339	33.8
Yes	663	66.2
Use of sleeping medication		
Never	992	99
< 1/week	7	0.7
1–2/week	2	0.2
≥ 3/week	1	0.1
Daytime dysfunction		
No	475	47.4
Yes	527	52.6
Total score		
≤ 5 (good sleep)	759	75.7
> 5 (poor sleep)	243	24.1

The PSQI total score of the infertile women was 7.53 ± 1.78, which is significantly higher than the Chinese norm (52). Except for sleep efficiency, all the components differed. Compared to the Chinese norm, the overall sleep quality, sleep latency, sleep duration, sleep disturbance, and daytime dysfunction in infertile women were significantly higher, whereas the use of sleeping medication was significantly lower (**Table 3**).

**Table 3:** Comparison of PSQI scores between infertile women (N = 1002) and Chinese norm.

Items	PSQI^a^ score			
	Infertile women	Normal subjects	t-value	p-value
Subjective sleep quality	0.72 ± 0.59	0.47 ± 0.53	13.42	< 0.001
Sleep latency	1.20 ± 1.23	0.70 ± 0.60	12.91	< 0.001
Sleep duration	0.38 ± 0.64	0.33 ± 0.55	2.62	0.009
Habitual sleep efficiency	0.31 ± 0.62	0.32 ± 0.63	–0.55	0.586
Sleep disturbances	0.69 ± 0.52	0.56 ± 0.52	7.80	< 0.001
Use of sleeping medication	0.01 ± 0.15	0.03 ± 0.20	–0.329	0.001
Daytime dysfunction	0.73 ± 0.82	0.12 ± 0.37	23.3	< 0.001
Total	4.04 ± 2.79	2.52 ± 1.60	17.26	< 0.001

a PSQI: Pittsburgh Sleep Quality Index

### Correlation analysis between the scores of psychological scales and sleep quality

The BDI, SAS, and PSS scores were significantly negatively correlated with PSQI total score, subjective sleep quality, sleep latency, sleep duration, habitual sleep efficiency, sleep disturbances, and daytime dysfunction scores (P < 0.01), but were not correlated with use of sleeping medication score (P > 0.05). CD-RISC score was significantly negatively correlated with PSQI total score, subjective sleep quality, sleep latency, habitual sleep efficiency, sleep disturbances, and daytime dysfunction scores (P < 0.01), but was not correlated with sleep duration, use of sleeping medication, and daytime dysfunction scores (P > 0.05, **Table 4**).

**Table 4:** Correlation between scores of different psychological scales and sleep quality in infertile women (N = 1002).

items	Subjective sleep quality	Sleep latency	Sleep duration	Habitual sleep efficiency	Sleep disturbances	Use of sleeping medication	Daytime dysfunction	Total
BDI^a^ score	0.278**	0.244**	0.132**	0.131**	0.275**	0.014	0.340**	0.378**
SAS^b^ score	0.291**	0.286**	0.160**	0.165**	0.297**	0.023	0.370**	0.427**
PSS^c^ score	0.183**	0.124**	0.088**	0.102**	0.154**	–0.014	0.190**	0.220**
CD-RISC^d^ score	–0.121**	–0.075*	–0.03	–0.111**	–0.078*	0.049	–0.06	–0.119**
F1^e^	–0.130**	–0.068*	–0.032	–0.094**	–0.085**	0.049	–0.081*	–0.123**
F2^f^	–0.105**	–0.071*	–0.031	–0.099**	–0.056	0.051	–0.048	–0.104**
F3^g^	–0.095**	–0.071*	–0.035	–0.122**	–0.069*	0.025	–0.033	–0.107**
F4^h^	–0.089**	–0.068*	–0.025	–0.068*	–0.084**	0.026	–0.068*	–0.104**
F5^i^	–0.0758*	–0.043	0.015	–0.099**	–0.019	0.061	–0.01	–0.056
PANAS^j^ (Positive Affect)	0.046	0.054	–0.007	–0.028	0.063*	0.000	0.039	0.049
PANAS^j^ (Negative Affect)	0.056	0.055	0.000	–0.035	0.053	0.01	0.044	0.052

a BDI: Beck Depression Inventory.

b SAS: Self-rating anxiety scale.

c PSS: Perceived Stress Scales.

d CD-RISC: Connor-Davidson resilience scale.

e F1: Factor 1 reflects the notion of personal competence, high standards, and tenacity.

f F2: Factor 2 corresponds to trust in one’s instincts, tolerance of negative affect, and strengthening effects of stress.

g F3: Factor 3 relates to the positive acceptance of change and secure relationships.

h F4: Factor 4 relates to control.

i F5: Factor 5 to spiritual influences.

j PANAS: Positive and Negative Affect Scale.

*represents P<0.05;** represents P<0.01.

### Influencing factors of sleep quality

We further studied the relationship between demographics and fertility characteristics, psychological characteristics, and sleep quality. A multiple logistic stepwise regression model was constructed, with sleep quality as dependent variable, age, BMI, education level, household income, cycle type, infertility duration, infertility type, cause of infertility, previous pregnancy history, number of previous treatment cycles, BDI, SAS, PSS, CD-RISC, positive affect, and negative affect scores as independent variables. We found that infertility type, BDI, SAS, PSS, and positive affect scores predicted the sleep quality of infertile women (**Table 5**).

**Table 5:** Multiple logistic regression analyses of influencing factors of sleep quality (N = 1002).

Variables	B	SE	OR (95% CI)	p-value
Infertility type	–0.418	0.17	0.66 (0.47, 0.92)	0.014
BDI score	0.097	0.026	1.10 (1.05, 1.16)	< 0.001
SAS score	0.133	0.018	1.42 (1.10, 1.18)	< 0.001
PSS score	0.023	0.011	1.02 (1.00, 1.05)	0.04
Positive Affect score	–0.036	0.018	0.97 (0.93, 1.00)	0.046

### Associations between sleep characteristics and IVF/ICSI outcomes

The relationship between sleep characteristics and intermediate reproductive outcomes is shown in **Table 6**. Collinearity diagnostics indicated no serious multicollinearity among variables (VIF < 5) and the model is well constructed. After adjusting for potential influencing factors including BMI, IVF/ICSI treatment cycle, duration of infertility, cause of infertility, and type of infertility, the number of oocytes retrieved (IRR= 0.85, 95% CI: 0.72–0.99; P = 0.038) and the number of mature oocytes (IRR = 0.82, 95% CI: 0.69–0.97; P = 0.022) in women with poor subjective sleep quality were significantly lower than in women with good subjective sleep quality. The fertilization rate (IRR = 0.96, 95% CI: 0.92–1.00; P = 0.034) was significantly lower in women with sleep disturbance compared with women without sleep disturbance. Compared with women who slept 7 to 8 hours per night, the oocyte retrieval rate (IRR= 1.04, 95% CI: 1.00–1.09; P = 0.031) of women who slept less than 7 hours per night was significantly higher.

**Table 6:** Associations between sleep characteristics and IVF/ICSI intermediate reproductive outcomes (N = 893)^a^.

Characteristics	Number of oocytes retrieved	Oocyte retrieval rate	Number of mature oocytes	Fertilization rate	Number of good-quality embryos
Subjective sleep quality					
Very good & fairly good	Ref	Ref	Ref	Ref	Ref
Very bad & fairly bad	**0.85 (0.72, 0.99)**	0.97 (0.91, 1.03)	**0.82 (0.69, 0.97)**	1.03 (0.95, 1.12)	0.85 (0.67, 1.07)
Trouble falling asleep (times/week)					
Never	Ref	Ref	Ref	Ref	Ref
< 1/week	0.97 (0.88, 1.07)	0.98 (0.96, 1.01)	0.97 (0.88, 1.08)	1.01 (0.96, 1.05)	0.94 (0.81, 1.10)
1–2/week	0.93 (0.79, 1.10)	0.96 (0.92, 1.02)	0.92 (0.78, 1.09)	0.97 (0.91, 1.04)	0.86 (0.68, 1.08)
≥ 3/week	1.06 (0.87, 1.29)	0.99 (0.93, 1.06)	1.08 (0.87, 1.34)	1.04 (0.95, 1.15)	1.15 (0.89, 1.49)
Sleep duration					
< 7 h	1.11 (0.96,1.29)	**1.04 (1.00, 1.09)**	1.11 (0.95, 1.30)	0.97 (0.90, 1.04)	1.11 (0.90, 1.38)
7 to < 8 h	Ref	Ref	Ref	Ref	Ref
8 to < 9 h	0.94 (0.86, 1.03)	1.01 (0.98, 1.04)	0.94 (0.86, 1.04)	0.99 (0.95, 1.03)	0.96 (0.84, 1.09)
9 to < 10 h	0.95 (0.84, 1.07)	1.03 (1.00, 1.06)	0.94 (0.83, 1.07)	1.02 (0.97, 1.08)	0.92 (0.77, 1.09)
≥ 10 h	0.90 (0.75, 1.07)	1.02 (0.98, 1.07)	0.91 (0.75, 1.10)	0.98 (0.91, 1.06)	0.96 (0.74, 1.24)
Habitual sleep efficiency					
≥ 85%	Ref	Ref	Ref	Ref	Ref
< 85%	0.94 (0.86, 1.04)	0.99 (0.96, 1.02)	0.95 (0.86, 1.05)	1.00 (0.96, 1.05)	0.90 (0.79, 1.02)
Sleep disturbance					
No	Ref	Ref	Ref	Ref	Ref
Yes	1.06 (0.97, 1.15)	1.01 (0.99, 1.03)	1.04 (0.95, 1.14)	**0.96 (0.92, 1.00)**	1.01 (0.90, 1.14)
Daytime dysfunction					
No	Ref	Ref	Ref	Ref	Ref
Yes	0.96 (0.89, 1.05)	1.01 (0.99, 1.03)	0.97 (0.89, 1.06)	1.02 (0.99, 1.06)	1.02 (0.91, 1.14)

a Models were adjusted for age, BMI, duration of infertility, infertility type, cause of infertility, previous pregnancy, and number of previous IVF/ICSI cycles. Ref, reference.

The bold values were confidential intervals of variables with p values < 0.05.

The relationship between sleep characteristics and clinical pregnancy is shown in **Table 7**. Collinearity diagnostics indicated no serious multicollinearity among variables(VIF < 5) and the model is well constructed. After adjusting for potential influencing factors including age, BMI, IVF/ICSI treatment cycle, duration of infertility, cause of infertility, and type of infertility, the clinical pregnancy rate (OR = 0.51, 95% CI: 0.29–0.88; P = 0.016) of women with sleep efficiency < 85% was significantly reduced compared to that of women with sleep efficiency ≥ 85%. In addition, after excluding women with night shift experience, we re-analyzed the relationship between sleep characteristics and IVF/ICSI outcomes and found similar results (**Supplementary Tables 1**, **2**).

**Table 7:** Associations between sleep characteristics and IVF/ICSI clinical pregnancy (N = 404)^a^.

Characteristics	Clinical pregnancy
Subjective sleep quality	
Very good & fairly good	Ref
Very bad & fairly bad	1.44 (0.54, 3.88)
Trouble falling asleep (times/week)	
Never	Ref
< 1/week	1.41 (0.79, 2.51)
1–2/week	1.20 (0.53, 2.70)
≥ 3/week	0.72 (0.20, 2.58)
Sleep duration	
< 7 h	1.74 (0.64, 4.75)
7 to < 8 h	Ref
8 to < 9 h	0.68 (0.40, 1.15)
9 to < 10 h	0.69 (0.33, 1.43)
≥ 10 h	0.69 (0.26, 1.81)
Habitual sleep efficiency	
≥ 85%	Ref
< 85%	**0.51 (0.29, 0.88)**
Sleep disturbances	
No	Ref
Yes	0.68 (0.41, 1.11)
Daytime dysfunction	
No	Ref
Yes	0.94 (0.59, 1.48)

a Models were adjusted for age, BMI, duration of infertility, infertility type, cause of infertility, previous pregnancy, and the number of previous IVF/ICSI cycles. Ref, reference.

The bold values were confidential intervals of variables with p values < 0.05.

## Discussion

Our study provides a comprehensive assessment of sleep characteristics in the early stage of IVF/ICSI treatment, explores the effects of negative and positive psychological factors on sleep quality, and elucidates the relationship between sleep characteristics and multiple IVF/ICSI treatment outcomes within a large sample. Over one-quarter of the participants reported poor sleep. Logistic regression analysis of sleep quality indicated that infertility type, depression, anxiety, perceived stress, and positive affect influenced sleep quality. Among them, depression, anxiety, and perceived stress were risk factors, positive affect was a protective factor for sleep quality, and psychological resilience could not predict sleep quality. The generalized linear model analysis showed that poor subjective sleep quality was negatively correlated with the number of retrieved oocytes and mature oocytes.

We found that 243 (24.1%) participants had poor sleep quality before IVF/ICSI treatment. Approximately 10% of the participants reported sleep latency longer than 30 minutes, 8.2% reported sleep duration less than 7 hours, and 23.9% reported less than 85% sleep efficiency. The incidence of poor sleep in this study was similar to the results of Yang’s (53) and Reschini’s (52) studies. Sleep quality may fluctuate with treatment cycles and measurement criteria. Factors such as medication and reproductive hormone changes during treatment may affect sleep quality (7, 29, 32, 33, 54), resulting in a different incidence of poor sleep in different treatment stages. Approximately 20%–60% of infertile women report sleep disturbances at various stages of treatment based on the criteria of poor sleep by PSQI > 5 (29, 30, 53, 55, 56). A study based on PSQI ≥ 6 as criteria for poor sleep found that 23% and 46% of infertile women had poor sleep during oocyte extraction and embryo transfer, respectively (31). Females with poor sleep are more likely to develop neuroendocrine disorders (57), resulting in decreased ovarian function (58), irregular menstruation (59), and reduced chances of conception (9). Moreover, preoperative sleep deprivation may lead to increased pain sensitivity (60), which will aggravate patients’ pain during IVF/ICSI treatment. Therefore, medical staff should focus more on the patient’s sleep condition and provide treatment for sleep problems.

In addition, compared to the Chinese norm, total PSQI score, subjective sleep quality, sleep latency, sleep duration, sleep disturbances, and daytime dysfunction of infertile women before ART treatment were significantly higher, whereas the use of sleeping medication was significantly lower. In addition to the fluctuation of sex hormones, financial burden, mental stress associated with ART, and concerns about pregnancy failure may contribute to poor sleep (32, 33, 38, 53, 54). Infertile women before IVF/ICSI treatment are less likely to use sleep medication than the general population, possibly due to concerns about conflict with fertility medication (30). Medical care staff could consider using psychological therapies to improve the sleep quality of these patients, such as cognitive behavioral therapy, physical and mental relaxation therapy, music therapy, etc. (61–63).

In this study, 23.2% of infertile women suffered from depression, 4.1% from anxiety, and 25.3% from health risk stress. In our study, the prevalence of depression and anxiety was lower than that of Huang et al. (30). This may be due to the different stages of data collection (before ovulation induction treatment *vs*. receiving hormonal stimulation phase). Both the diagnosis and treatment of infertility are important stressors (64–66). This process may cause feelings of stress, anxiety, depression, hopelessness, guilt, etc. (65, 67–70). Lund believes that emotions affect sleep (34). Multivariate logistic regression analysis showed that infertility type, depression, anxiety, perceived stress, and positive affect significantly impacted sleep quality. Women with secondary infertility have a lower risk of sleep disturbances than those with primary infertility. Previous studies showed that women with primary infertility were significantly more stressed (71) and depressed (72) than women with secondary infertility, whereas their subjective well-being was similar (73). The possible reason is that women with primary infertility have never been pregnant and are more psychologically stressed. In summary, depression, anxiety, and perceived stress in infertile women were associated with poor sleep quality. This is consistent with previous studies (30, 42, 74). Individuals with high positive affect tended to report better sleep quality, similar to the findings of Fortunato et al. (75). It should be noted that the perceived stress and positive affect yielded p-values close to 0.05. More caution is needed when interpreting the results. In addition, a comparison between participants with poor and good sleep quality showed that a higher BMI was related to worse sleep quality, this is consistent with the results from a large-scale prospective study conducted in 2366 pre-pregnant women (76).

Our study found that sleep characteristics were associated with IVF/ICSI clinical outcomes. Subjective sleep quality negatively correlated with the number of retrieved and mature oocytes. Sleep disturbance negatively correlated with fertilization rate. Consistent with our results, Huang et al. reported that women with poor sleep quality had decreased oocyte quality and fertilization rate (30). Llaneza et al. found that sleep disorders were associated with decreased oocyte retrieval and poor ovarian response in a study of 200 Spanish women (58). Our study also found that women with sleep efficiency < 85% had a reduced clinical pregnancy rate compared with women with sleep efficiency ≥ 85%, supporting previous studies. Previous studies reported that long or short nocturnal sleep was associated with a reduced clinical pregnancy rate (32, 52, 77, 78). Liu et al. found that good sleep quality positively correlated with clinical pregnancy and live birth rates (78). In addition, our study found an association between sleep duration and the rate of retrieved oocytes but not for other outcomes. Previous studies in the general population have found that short sleep duration is associated with abnormal menstruation, abnormal reproductive hormone level, and decreased fertility (59, 79, 80). However, there have been limited and conflicting studies on the relationship between sleep duration and IVF/ICSI treatment outcomes among women undergoing infertility treatment. One study found that short sleep duration (< 7 h) was associated with decreased oocyte quantity and quality, and long sleep duration (9–10 h) was associated with a decreased chance of pregnancy (81). A study of 656 women undergoing IVF found that moderate sleep duration (7–8 h) was associated with a higher pregnancy rate than long sleep duration (9–11 h) and short sleep duration (4–6 h). However, no correlation was found between sleep duration and the number of retrieved oocytes or the fertilization rate (77). Our study found that short sleep duration (< 7 h) had a higher rate of retrieved oocytes than moderate sleep duration (7–8 h). The inconsistencies between studies may be related to study design (retrospective *vs*. prospective study) and the sleep assessment time points (before ovulation induction treatment *vs*. on the day of oocyte retrieval), and differences in study populations (78, 81). Therefore, multiple assessments and longitudinal design studies must confirm the association between sleep duration before treatment and reproductive outcomes.

Several limitations should be considered. First, the measurement of sleep quality was based on subjective scales and lacked objective data support. Second, all the participants were recruited from Shandong Province, China. Thus, our results may not apply to all infertile women in China. Third, we did not track the dynamic changes in sleep quality of infertile women at different stages of ART treatment. Fourth, although we adjusted for several confounding factors in the statistical analysis, other covariates may have confounded the results, such as treatment plans and lifestyle. Last, we did not correct for multiple comparisons. The influence of perceived stress and positive affect on sleep quality may need further replication. Therefore, future studies may benefit from including objective measures such as actigraphy or polysomnography and expanding the sample range.

## Conclusion

In this study, we found that the sleep quality of infertile women before ovulation induction was poorer than that of normal adults. Primary infertility, depression, anxiety, and stress are risk factors, while positive affect is a protective factor for poor sleep quality. Our findings have significant implications for public health and reveal that assessing sleep conditions and improving sleep quality before ART treatment may benefit treatment outcomes.

## Data availability statement

The original contributions presented in the study are included in the article/**Supplementary Material**. Further inquiries can be directed to the corresponding authors.

## Ethics statement

The studies involving humans were approved by Medical Science Ethics Board of Affiliated Hospital of Jining Medical University, China. The studies were conducted in accordance with the local legislation and institutional requirements. The participants provided their written informed consent to participate in this study.

## Author contributions

Y-XX, CW, and LZ contributed to the conception and design of the study. LZ and Y-SX recruited the participants. LZ collected the data. K-XC, Q-LL, and LC organized the database. Q-LL performed the statistical analysis. K-XC and Q-LL wrote the first draft of the manuscript. All authors contributed to manuscript revision, read, and approved the submitted version. Y-XX administrated the projection.
