# Supplementary material for: Sleep characteristics before assisted reproductive technology treatment predict reproductive outcomes: a prospective cohort study of Chinese infertile women
Source: Front Endocrinol (Lausanne). 2023 Oct 16;14:1178396. doi: 10.3389/fendo.2023.1178396 (PMC10614022; doi:10.3389/fendo.2023.1178396)
Supplement: Supplementary file 1 [file Table_1.docx]

**Supplementary Table 1.** Associations between sleep characteristics with IVF/ICSI intermediate reproductive outcomes after excluding women with night shift experience (N = 838).

| Characteristics | Number of oocytes retrieved | Oocytes retrieved rate | Number of mature oocytes | Fertilization rate | Number of good-quality embryos |
| --- | --- | --- | --- | --- | --- |
| Subjective sleep quality | | | | | |
| Very good & fairly good | Ref | Ref | Ref | Ref | Ref |
| Very bad & fairly bad | **0.84 (0.71, 0.99)** | 0.96 (0.90, 1.02) | **0.81 (0.68, 0.97)** | 1.03 (0.95,1.12) | 0.81 (0.63, 1.04) |
| Trouble falling asleep (times/week) | | | | | |
| Never | Ref | Ref | Ref | Ref | Ref |
| < 1/week | 0.96 (0.86, 1.06) | 0.98 (0.95, 1.01) | 0.96 (0.86, 1.08) | 1.01 (0.96, 1.05) | 0.95 (0.81, 1.11) |
| 1-2/week | 0.92 (0.78, 1.09) | 0.96 (0.91, 1.02) | 0.92 (0.77, 1.09) | 0.97 (0.91, 1.04) | 0.87 (0.68, 1.11) |
| ≥ 3/week | 1.05 (0.86, 1.29) | 0.99 (0.93, 1.06) | 1.08 (0.87, 1.34) | 1.04 (0.95, 1.15) | 1.19 (0.91, 1.55) |
| Sleep duration |  |  |  |  |  |
| < 7 h | 1.09 (0.94, 1.27) | **1.05 (1.01, 1.09)** | 1.09 (0.93, 1.28) | 0.97 (0.90, 1.04) | 1.09 (0.87, 1.36) |
| 7 to < 8 h | Ref | Ref | Ref | Ref | Ref |
| 8 to < 9 h | 0.92 (0.84, 1.02) | 1.01 (0.98, 1.04) | 0.93 (0.84, 1.03) | 0.99 (0.95, 1.03) | 0.94 (0.82, 1.08) |
| 9 to < 10 h | 0.95 (0.84, 1.08) | 1.03 (1.00, 1.06) | 0.94 (0.82, 1.08) | 1.02 (0.97, 1.08) | 0.92 (0.76, 1.10) |
| ≥ 10 h | 0.86 (0.72, 1.02) | 1.02 (0.98, 1.07) | 0.86 (0.72, 1.04) | 0.98 (0.91, 1.06) | 0.89 (0.70, 1.14) |
| Habitual sleep efficiency | | | | | |
| ≥ 85% | Ref | Ref | Ref | Ref | Ref |
| < 85% | 0.96 (0.87, 1.06) | 0.99 (0.96, 1.02) | 0.97 (0.88, 1.07) | 1.00 (0.96, 1.00) | 0.92 (0.81, 1.05) |
| Sleep disturbances | | | | | |
| No | Ref | Ref | Ref | Ref | Ref |
| Yes | 1.06 (0.97, 1.16) | 1.01 (0.99, 1.04) | 1.04 (0.95, 1.14) | **0.96 (0.92, 1.00)** | 1.00 (0.89, 1.13) |
| Daytime dysfunction |  |  |  |  |  |
| No | Ref | Ref | Ref | Ref | Ref |
| Yes | 0.98 (0.90, 1.07) | 1.01 (0.99, 1.04) | 0.99 (0.91, 1.09) | 1.02 (0.99, 1.06) | 1.04 (0.92, 1.17) |

^a^Models were adjusted for age, BMI, duration of infertility, infertility type, cause of infertility, previous pregnancy, and number of previous IVF/ICSI cycles. Ref, reference.
