# Supplementary material for: Sleep characteristics before assisted reproductive technology treatment predict reproductive outcomes: a prospective cohort study of Chinese infertile women
Source: Front Endocrinol (Lausanne). 2023 Oct 16;14:1178396. doi: 10.3389/fendo.2023.1178396 (PMC10614022; doi:10.3389/fendo.2023.1178396)
Supplement: Supplementary file 2 [file Table_2.docx]

**Supplementary Table 2.** Associations of sleep characteristics with IVF/ICSI clinical pregnancy after excluding women with night shift experience (N = 349)^a^.

| Characteristics | Clinical pregnancy |
| --- | --- |
| Subjective sleep quality |  |
| Very good & fairly good | Ref |
| Very bad & fairly bad | 1.53 (0.54, 4.39) |
| Trouble falling asleep (times/week) |  |
| Never | Ref |
| < 1/week | 1.36 (0.75, 2.45) |
| 1-2/week | 1.13 (0.50, 2.58) |
| ≥ 3/week | 0.66 (0.18, 2.47) |
| Sleep duration |  |
| < 7 h | 2.01 (0.71, 5.73) |
| 7 to < 8 h | Ref |
| 8 to < 9 h | 0.65 (0.38, 1.11) |
| 9 to < 10 h | 0.66 (0.31, 1.40) |
| ≥ 10 h | 0.62 (0.23, 1.69) |
| Habitual sleep efficiency |  |
| ≥ 85% | Ref |
| < 85% | **0.51 (0.29, 0.89)** |
| Sleep disturbances |  |
| No | Ref |
| Yes | 0.69 (0.42, 1.15) |
| Daytime dysfunction |  |
| No | Ref |
| Yes | 1.03 (0.64, 1.65) |

^a^Models were adjusted for age, BMI, duration of infertility, infertility type, cause of infertility, previous pregnancy, and the number of previous IVF/ICSI cycles. Ref, reference.
